# Covalent modification of Keap1 at Cys77 and Cys434 by pubescenoside a suppresses oxidative stress-induced NLRP3 inflammasome activation in myocardial ischemia-reperfusion injury

**DOI:** 10.7150/thno.48436

**Published:** 2021-01-01

**Authors:** Yuanyuan Cheng, Liangkai Cheng, Xiang Gao, Sixuan Chen, Peng Wu, Caiyan Wang, Zhongqiu Liu

**Affiliations:** 1 Guangdong Key Laboratory for Translational Cancer Research of Chinese Medicine, Joint Laboratory for Translational Cancer Research of Chinese Medicine of the Ministry of Education of the People's Republic of China, International Institute for Translational Chinese Medicine, School of Pharmaceutical Sciences, Guangzhou Univ Chinese Med, Guangzhou, Guangdong, 510006, China; 2 School of Pharmaceutical Sciences, Xiamen University, Xiamen, Fujian, 361102, China

**Keywords:** pubescenoside A, myocardial ischemia-reperfusion injury, Kelch ECH-associating protein 1, nuclear factor erythroid 2-related factor 2, covalent modification

## Abstract

**Background and Purpose:** Kelch ECH-associating protein 1 (Keap1) is a crucial chaperonin for E3 ubiquitin ligases. Modification of the key reactive cysteine residues in Keap1 affects the interaction between Keap1 and its substrate nuclear factor erythroid 2-related factor 2 (Nrf2), subsequently regulating oxidative stress and NLPR3 inflammasome activation, which are important factors for myocardial ischemia-reperfusion injury (MI/RI). Pubescenoside A (PBA), an active compound from *Ilex pubescens,* has antithrombotic and anti-inflammatory effects. However, the effect of PBA on MI/RI is still unknown. In the present study, we aimed to determine whether PBA can protect the heart against MI/RI and clarify the direct target and the underlying mechanism of PBA.

**Methods:** The left anterior descending artery (LAD) ligation-induced MI/RI mice model or oxygen and glucose deprivation/reperfusion (OGD/R) were used to evaluate the cardioprotective effect of PBA. Pull-down assays, co-immunoprecipitation (Co-IP) assays, LC/MS/MS, isothermal calorimetry (ITC) experiments and covalent docking were used to identify the target of PBA.

**Results:** PBA protected cardiomyocytes against OGD/R *in vitro* and LAD-induced MI/RI *in vivo*. PBA suppressed NLRP3 inflammation activation and induced the Nrf2 signaling pathway. Interestingly, PBA targeted Keap1 by selectively covalently binding to conserved cysteine residues, cysteine 77 (Cys77) in the BTB domain and cysteine 434 (Cys434) in the Kelch domain of Keap1, subsequently inhibiting ubiquitination of Nrf2 and activating antioxidant enzymes. Additionally, the cysteines of Keap1 has different degree of activation by PBA as follows: Cys77 > Cys434 > Cys23 > Cys38 > Cys226 > Cys273, which further elucidates the cysteine sensitivity of Keap1.

**Conclusions:** Our results indicated that PBA might be a new Nrf2 activator that covalently binds to two critical domains of Keap1, and shows cardioprotective activities against ischemia-reperfusion injury.

## Introduction

Kelch ECH-associating protein 1 (Keap1) is a crucial chaperonin for E3 ubiquitin ligases 1. Keap1 has five domains: the NTR, BTB, IVR, Kelch (DGR) and CTR domains 2. Cysteines play important roles in the on-off switch for the functions of Keap1; for example, Cys151, Cys273, and Cys288 are well recognized as the key cysteines for Keap1 3, 4. Keap1 has several partners, such as P53-induced protein with a death domain (PIDD), nuclear factor erythroid 2-related factor 2 (Nrf2) and p62 5, 6. The BTB domain and Kelch domain can regulate Nrf2 ubiquitination and degradation 7, 8. Nuclear factor erythroid 2-related factor 2 (Nrf2) is a transcription factor and sensitive to redox potential 9. It induces several antioxidative proteins and antioxidants to suppress reactive oxygen species (ROS) 10, 11. Recent studies have revealed that several small molecules could modify these cysteines and affect the interaction between Keap1 and Nrf2 12, 13. However, few of them are being evaluated in clinical trials, because the activation and selection of these cysteines by the molecules are unclear.

Myocardial infarction is the leading cause of cardiovascular disease-related death worldwide 14. Reperfusion is an accepted therapy for myocardial infarction 15. However, reperfusion can increase the infarct size by 50% at some time, because myocardial ischemia-reperfusion (MI/R) initiates oxidative stress and inflammation, which aggravates the injury 16, 17. The NLRP3 inflammasome controls interleukin-1β (IL-1β) and IL-18 induction. Interestingly, inhibition of the NLPR3 inflammasome decreases cardiac infarct size, ameliorates adverse cardiac remodeling, and improves cardiac function in an animal model of MI/R 18, 19. Excessive ROS triggers the activation of the NLRP3 inflammasome 20, 21. ROS scavengers or knockdown of NADPH oxidase can inhibit the NLRP3 inflammasome 20, 22. Therefore, modification of cysteines on Keap1 to affect the Nrf2-related antioxidative signaling pathway might ameliorate NLRP3 inflammasome-related myocardial ischemia reperfusion.

**Figure 1:**
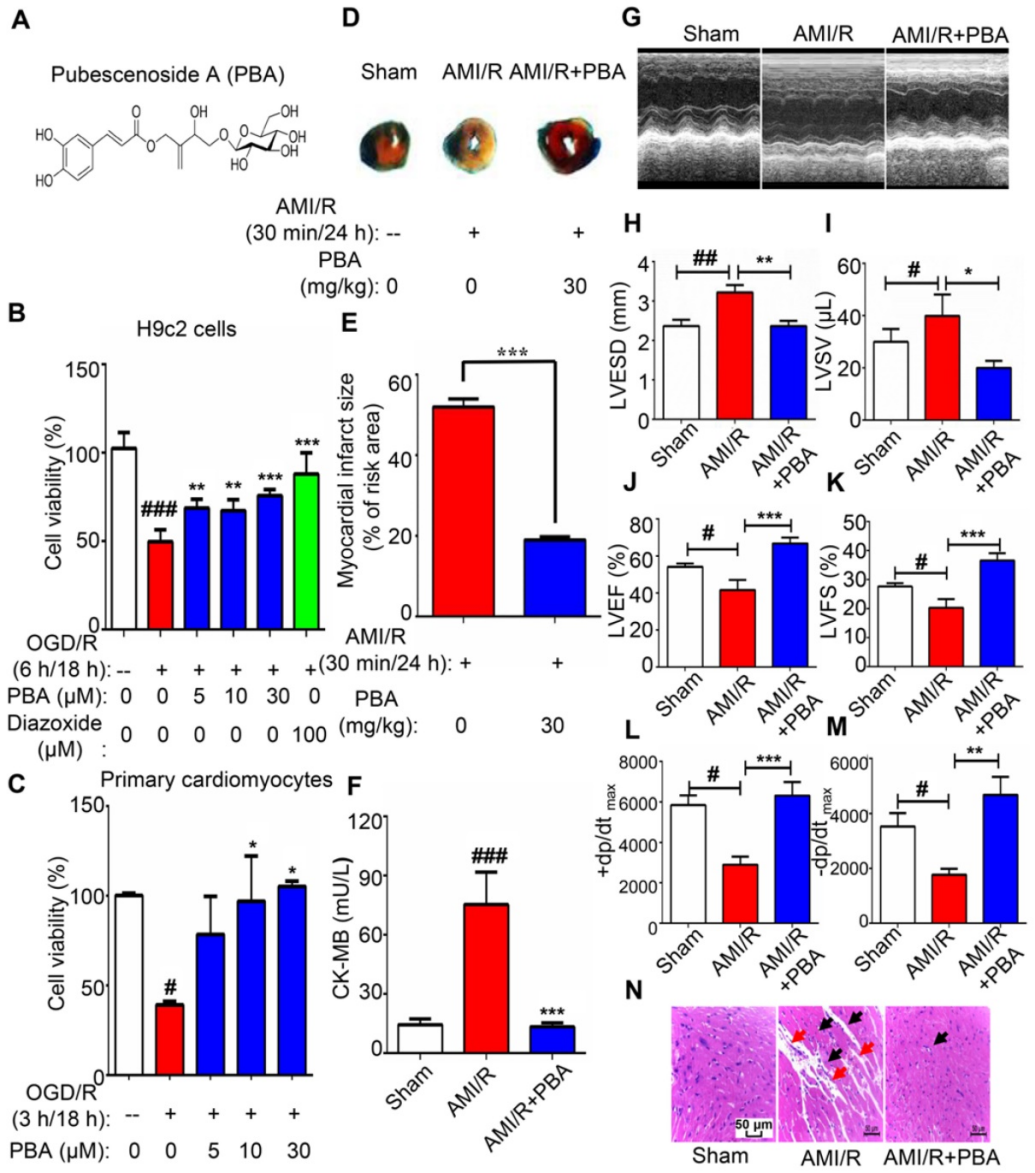
** PBA protected cardiomyocytes against ischemia-reperfusion.** (A) Chemical structure of PBA. (B and C) The effect of PBA on the OGD/R-induced H9c2 cells and primary mouse cardiomyocytes. H9c2 cells and primary mouse cardiomyocytes were cultured in 96-well plates. After removal of complete DMEM, DMEM without glucose and serum was added, and the cells were treated with different concentrations of PBA. The cells were cultured under OGD conditions as described in the Method section for 6 h and under normal conditions for another 18 h. The results are normalized to expressed as the mean ± SD (n = 5). ###*p* < 0.001, the control vs OGD/R; **p < 0.01, ***p < 0.001, OGD/R+PBA or OGD/R+diazoxide vs OGD/R. (D and E) The effect of PBA on myocardial ischemia-reperfusion injury in a mouse model. Detection and quantification of MI/R injury. After 30 min of ischemia and 24 h of reperfusion, the heart tissue sections were stained with 1% TTC and 1% Evans blue. The infract size was determined. The results are expressed as the mean ± SEM (n = 6) and data were analyzed by *t*-tests. ***p < 0.01, the AMI/R + PBA group vs the AMI/R group. (F) Level of cardiac CK-MB. After 30 min of ischemia and 24 h of reperfusion, the blood samples were collected and measured for CK-MB ELISA kit. Values represent the mean ± SEM (n = 6) and data were analysed by one-way ANOVA. ###*p* < 0.001, the sham group vs the AMI/R group; ***p < 0.001, the AMI/R +PBA group vs the AMI/R group. (G) Representative echocardiographic image under M-mode. (H-M) Analysis of left ventricular end systolic diameter (LVESD), left ventricular end systolic volume (LVSV), left ventricular ejection fraction (LVEF), left ventricular fractional shortening (LVFS), the maximum rate of rise of left ventricular pressure increase (+dp/dt_max_) and the maximum rate of rise of left ventricular pressure decrease (-dp/dt_max_) at 24 hours after AMI/R. #* p* < 0.05, ##*p* < 0.01, the sham group vs the AMI/R group; *p < 0.05, ** *p* < 0.01, *** *p* < 0.001, the AMI/R + PBA group vs the AMI/R group. (N) Histopathological examination of mouse hearts. The cardiac tissues were stained with H&E stain. Representative images were selected from three groups. The black arrows represent inflammatory cell infiltration, and the red arrows represent disrupted myofibrillar structure. Scale bar: 50 μm.

*Ilex pubescens* has been widely used in treating cardiovascular diseases in traditional Chinese medicine therapeutics 23-25. Our previous studies have demonstrated that pubescenoside A (PBA) is an active compound from *I. pubescens* (Fig. 1A), which has antithrombotic and anti-inflammatory effects 23. However, the underlying mechanism and the direct target for MI/R injury are still unknown.

During the early stages of AMI/R, the NLRP3 inflammasome specks can be detected in the endothelial cells, cardiomyocytes, and fibroblasts 16, 23. When the inflammatory cells infiltrate the heart, the majority of NLRP3 inflammasome specks can be found in macrophages 23. Moreover, cardiomyocytes injured are the major feature of AMI/R, and NLRP3 inflammasome activation in cardiomyocytes under oxygen-glucose deprivation and reperfusion (OGD/R) insult has been established 15. Thus, we chose cardiomyocytes and macrophages as representative cell types to study. In the present study, we first found that PBA could protect cardiomyocytes against oxygen-glucose deprivation and reperfusion injury *in vitro* and significantly ameliorate MI/R injury *in vivo*. Furthermore, PBA could block NLRP3 inflammasome activation. Importantly, our results demonstrated that PBA could covalently bind to the conserved Cys77 and Cys434 residues of Keap1, disturbing the interactions of the Cul3-Keap1 ubiquitin ligase complex and the Keap1-Nrf2 complex, subsequently activating the Nrf2 signaling pathway. Our findings revealed that PBA might be used as a lead compound for the discovery of new therapeutic drugs for the treatment of MI/R injury.

## Results

### PBA protects against myocardial ischemia-reperfusion injury

We initially evaluated the cytotoxicity of PBA on the growth of RAW264.7 macrophages, H9c2 cells and primary neonatal mouse cardiomyocytes using an MTT assay. As shown in Figure S1, PBA did not exhibit any cell toxicity under 240 μM in RAW264.7, H9c2 cells and primary cardiomyocytes. Thus, 30 μM or less of PBA was chosen in the following assays. The damage to cardiomyocytes in ischemic conditions is induced by reduction of glucose and oxygen. Oxygen and glucose deprivation/reperfusion (OGD/R) insult is recognized as a classic method to stimulate the pathological process of ischemia-reperfusion *in vitro*. Subsequently, we performed the OGD/R model in H9c2 cells and primary cardiomyocytes to investigate the cardioprotective effect of PBA.

After overnight culture, H9c2 cells and primary cardiomyocytes were exposed to 6 h or 3 h of OGD insult in the following experiments. H9c2 cells were treated with various doses of PBA and diazoxide (positive control drug) before being subjected to OGD. Then, the cells were reperfused and maintained for 18 h. Cell viability was detected by MTT assays. As shown in Figure 1B-C, PBA treatment (10 and 30 μM) protected H9c2 or primary cardiomyocytes against OGD/R induced injury. The protective effect of PBA seemed equal to that of 100 μM diazoxide.

Next, we used the LAD-induced myocardial ischemia-reperfusion model in mice to examine the cardioprotection of PBA. PBA administration at the dose of 30 mg/kg could significantly reduce myocardial ischemia-reperfusion induced cardiac infarct size and the level of CK-MB (Figure 1D-F). LVESD and LVSV enlarged after AMI/R, while LVEF, LVSF, +dp/dt_max_ and -dp/dt_max_ were decreased after AMI/R. However, PBA administration could improve cardiac function by increasing LVEF, LVFS, +dp/dt_max_ and -dp/dt_max_ and decreasing LVESD and LVSV (Figure 1G-M). H&E staining showed that AMI/R disrupted myofibrillar structure and caused neutrophil/macrophage clusters accumulation. PBA at the dose of 30 mg/kg not only preserved myofibrillar structure but also reduced the recruitment of inflammatory cells (Figure 1N).

### PBA suppresses activation of the NLRP3 inflammasome in macrophages, OGD/R-induced H9c2 cells and primary cardiomyocytes

**Figure 2:**
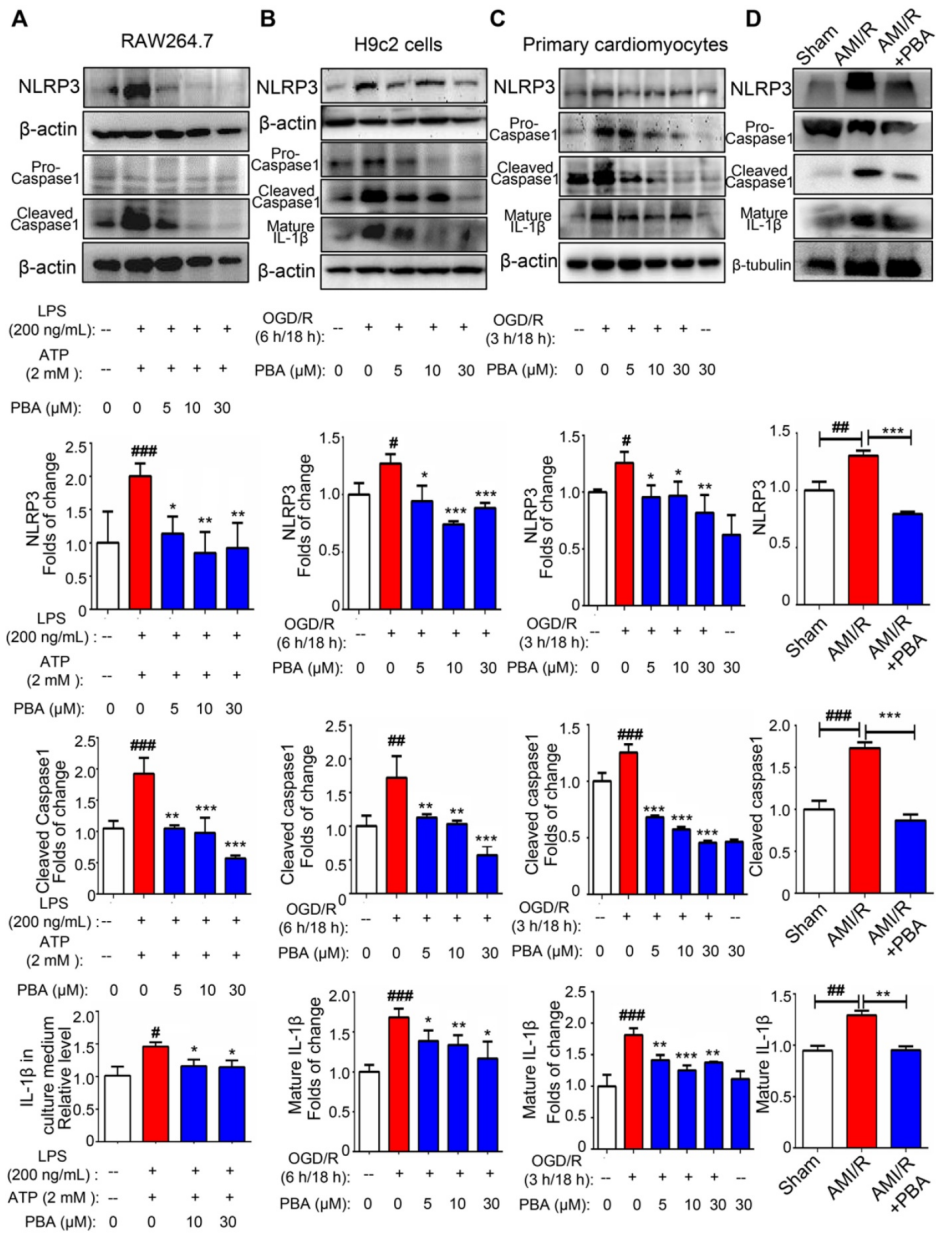
** PBA suppresses NLRP3 inflammasome activation.** (A) PBA inhibited NLRP3 inflammasome activation in RAW264.7 cells. The cells were pretreated with PBA at the indicated concentration for 1 h and then stimulated with 200 ng/mL LPS for 6 h. Then, 2 mM ATP was added to the culture medium for another 20 min. Protein was isolated and subjected to SDS-PAGE. Data are shown as the mean ± SD, n = 3. ###*p* < 0.001, the control vs LPS+ATP; *p < 0.05, **p < 0.01, LPS+ATP+PBA vs LPS+ATP. (B and C) The effect of PBA on NLRP3 inflammasome activation in OGD/R-induced H9c2 cells and primary cardiomyocytes. H9c2 cells or primary cardiomyocytes were subjected to OGD for 6 h or 3 h, and reperfusion for 18 h. Then, proteins were isolated for SDS-PAGE for analysis of NLRP3, cleaved-caspase1 and mature IL-1β. Data are shown as the mean ± SD, n = 3. #*p* < 0.05, ##*p* < 0.01, the control vs OGD/R; **p < 0.01, ***p < 0.001, OGD/R+PBA vs OGD/R. (D) The effect of PBA administration on NLRP3 inflammasome activation in heart tissue of AMI/R-induced mice. Data are shown as the mean ± SEM, n = 5. ##* p* < 0.01, ###*p* < 0.01, the sham group vs the AMI/R group; ** *p* < 0.01, *** *p* < 0.001, the AMI/R + PBA group vs the AMI/R group.

To evaluate the effect of PBA on the NLRP3 inflammasome, we first used an established method (LPS plus ATP) to induce the NLRP3 inflammasome in RAW264.7 cells. As shown in Figure 2A, PBA attenuated LPS/ATP-mediated NLPR3 induction as well as cleaved-caspase1 and IL-1β. And immunofluorescent staining in Figure S2 showed that PBA significantly reduced LPS/ATP-induced ACS and Caspase1 expression. To confirm the role of the PBA-mediated NLRP3 inflammasome in myocardial ischemia-reperfusion, we ascertained the efficacy of PBA in OGD/R-induced cardiomyocytes. Exposure of H9c2 cells and primary cardiomyocytes to OGD conditions (6 h or 3 h) followed by reperfusion (18 h) significantly increased the NLRP3, cleaved-caspase1 and mature IL-1β levels. However, OGD/R-induced inflammasome activation in cardiomyocytes was substantially inhibited by PBA in a dose-dependent manner (Figure 2B-C). In addition, as shown in Figure 2D, PBA administration also attenuated NLRP3 inflammasome activation in heart tissue of AMI/R-injured mice.

### PBA activates the Nrf2 pathway and induces protective enzymes

**Figure 3:**
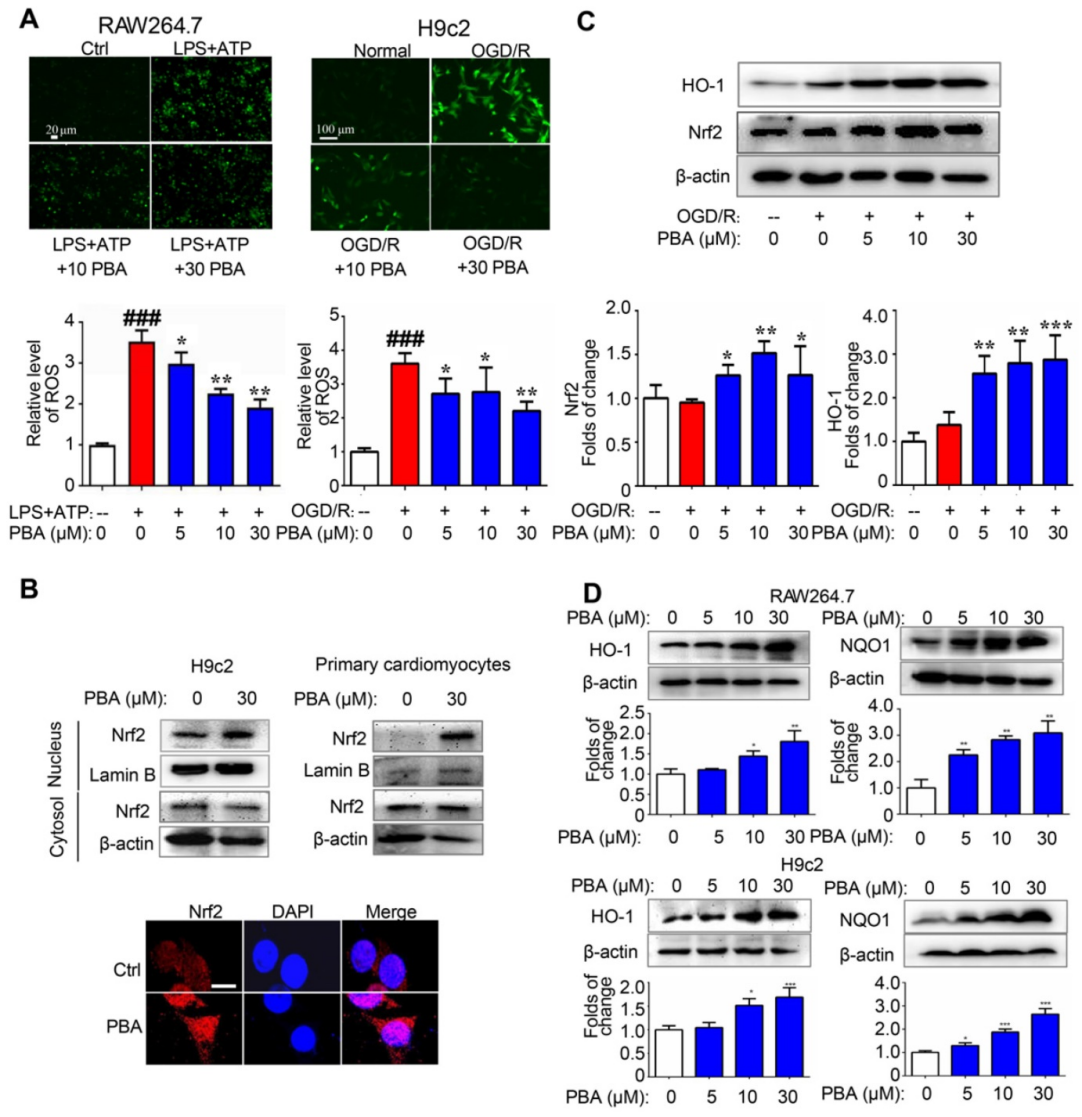
** PBA activates the Nrf2 signaling pathway.** (A) PBA decreased ROS production. Cells were treated with the indicated concentrations of PBA, and then stimulated with LPS plus ATP, and the ROS level was determined by the fluorescent probe DCFH-DA. The results are expressed as the mean ± SD (n = 3). (B) PBA promoted Nrf2 translocation into the nucleus. The cells were treated with PBA for 6 h. After isolation of the proteins in the cytosol and nucleus, the protein extract was subjected to SDS-PAGE for western blotting analysis. Other cells were used for Nrf2 immunofluorescence staining. Scale bar: 10 μm. (C) PBA increased Nrf2 expression and HO-1 expression in the OGD/R-induced cardiomyocytes. *p < 0.05, **p < 0.01, ***p < 0.001, OGD/R + PBA vs OGD/R. (D) The effects of PBA on HO-1 and NQO1 expression. Cells were treated with PBA for 24 h. *p < 0.05, **p < 0.01, PBA treatment vs the control.

**Figure 4:**
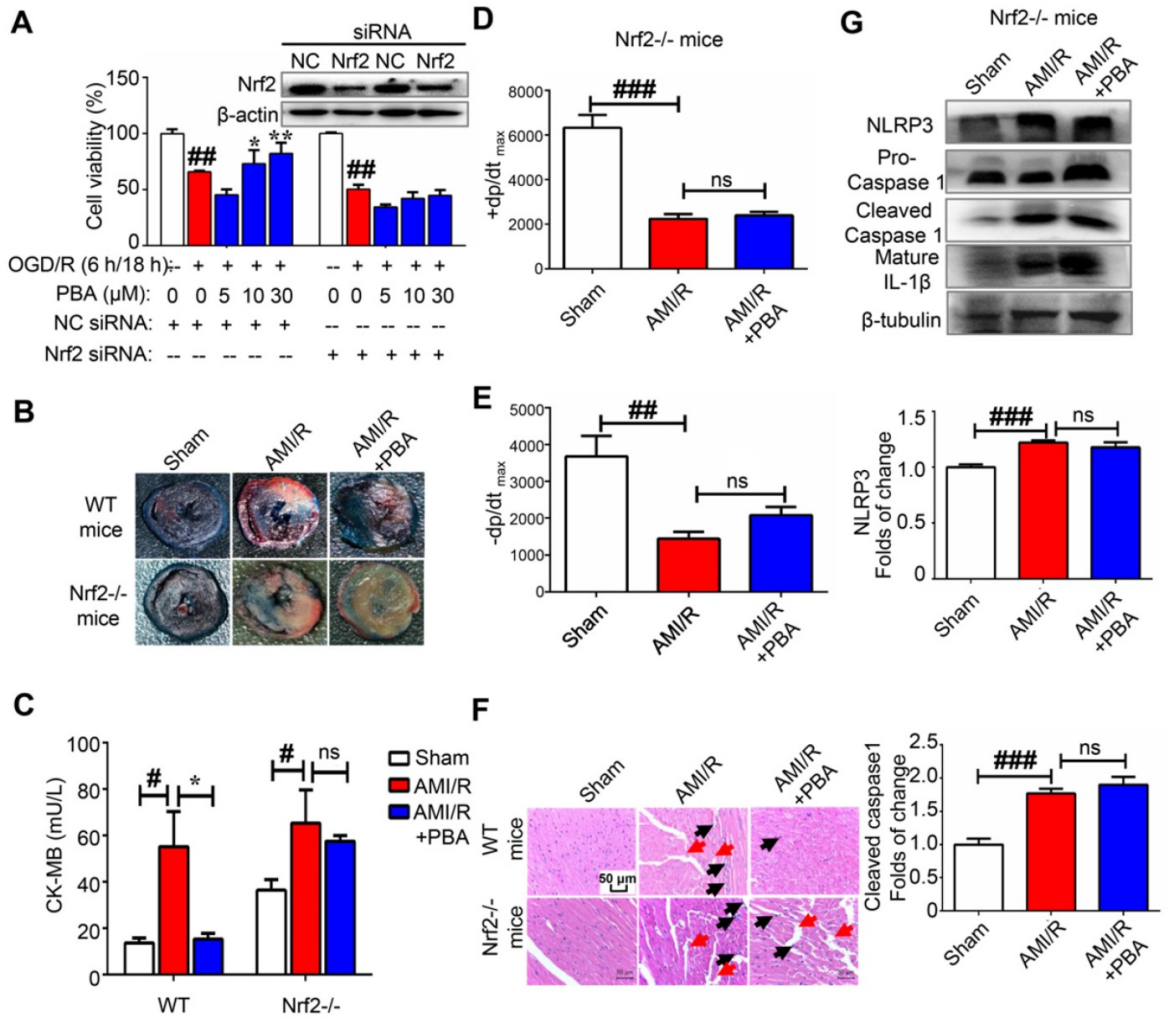
** PBA attenuates myocardial ischemia-reperfusion via the Nrf2 signaling pathway.** (A) PBA protected cardiomyocytes against OGD/R-induced cytotoxicity in a Nrf2-dependent manner. H9c2 cells were transfected with siNC and Nrf2 siRNA for 24 h. Then, the cells were treated with the indicated concentrations of PBA before OGD/R insult. Cell viability was measured by MTT assays. The results are expressed as the mean ± SD (n = 5). ##*p* < 0.01, OGD/R vs control; *p < 0.05, **p < 0.01, OGD/R vs OGD/R + PBA. (B) The effect of PBA on LAD-induced Nrf2-/- mice. (C) Level of cardiac CK-MB in Nrf2-/- mice. After 30 min of ischemia and 24 h of reperfusion, the blood samples were collected and measured for CK-MB ELISA kit. Values represent the mean ± SEM (n = 4) and data were analysed by one-way ANOVA. #*p* < 0.05, the sham group vs the AMI/R group; *p < 0.001, the AMI/R + PBA group vs the AMI/R group. (D and E) Analysis of the maximum rate of rise of left ventricular pressure increase (+dp/dt_max_) and the maximum rate of rise of left ventricular pressure decrease (-dp/dt_max_) at 24 hours after AMI/R in Nrf2-/- mice. #*#p* < 0.01, ###*p* < 0.001, the sham group vs the AMI/R group. (N) Histopathological examination of mouse hearts in Nrf2-/- mice. The cardiac tissues were stained with H&E stain. Representative images were selected from three groups. The black arrows represent inflammatory cell infiltration, and the red arrows represent disrupted myofibrillar structure. Scale bar: 50 μm. (D) The suppressive effect of PBA on the NLRP3 inflammasome was blocked in Nrf2-/- mice. After reperfusion, heart tissue protein was collected for WB analysis. Data are shown as the mean ± SEM, n = 5. ###*p* < 0.01, the sham group vs the AMI/R group.

ROS play an important role in the activation of the NLRP3 inflammasome. We found that PBA could significantly suppress the LPS plus ATP-induced ROS in macrophages and the OGD/R-induced ROS in H9c2 cells (Figure 3A). To explore the mechanism underlying the PBA-mediated reduction of ROS, we determined the effect of PBA on Nrf2 nuclear translocation. PBA significantly promoted Nrf2 translocation into nucleus (Figure 3B). Moreover, as shown in Figure 3C, PBA strongly induced Nrf2 expression in cardiomyocytes under OGD/R condition. Nrf2, as a transcription factor, induces the expression of a panel of antioxidant enzymes, such as heme oxygenase-1 (HO-1), and NAD(P)H quinone oxidoreductase 1 (NQO1). As shown in Figure 3D, PBA increased HO-1 and NQO1 expression at the protein level in a dose-dependent manner in both RAW264.7 and H9c2 cells. In addition, to investigate whether the cardioprotective effect of PBA is associated with Nrf2, we used Nrf2 specific small interfering RNA to knock down the expression of Nrf2. The results in Figure 4A show that H9c2 cells were more sensitive to OGD/R-induced injury after Nrf2 siRNA transfection. Moreover, the cardioprotection of PBA was blocked in the Nrf2 knockdown H9c2 cells. Consistent with* in vitro* study, the cardioprotective effect of PBA on Nrf2-/- mice under AMI/R injury was disappeared. Specifically, the decrease effect of CK-MB level and the increase effect of -dp/dt_max_ and -dp/dt_max_ by PBA were blocked in Nrf2-/- mice under AMI/R injury (Figure 4B-E). H&E staining in Figure 4F shows that PBA administration in Nrf2-/- mice could not suppress inflammatory cells infiltration and improve disturbed myofibrillar structure. These data suggested that Nrf2 was involved in the cardioprotective and NLRP3-suppressive effects of PBA.

### PBA directly targets Keap1 at Cys77 and Cys434

**Figure 5:**
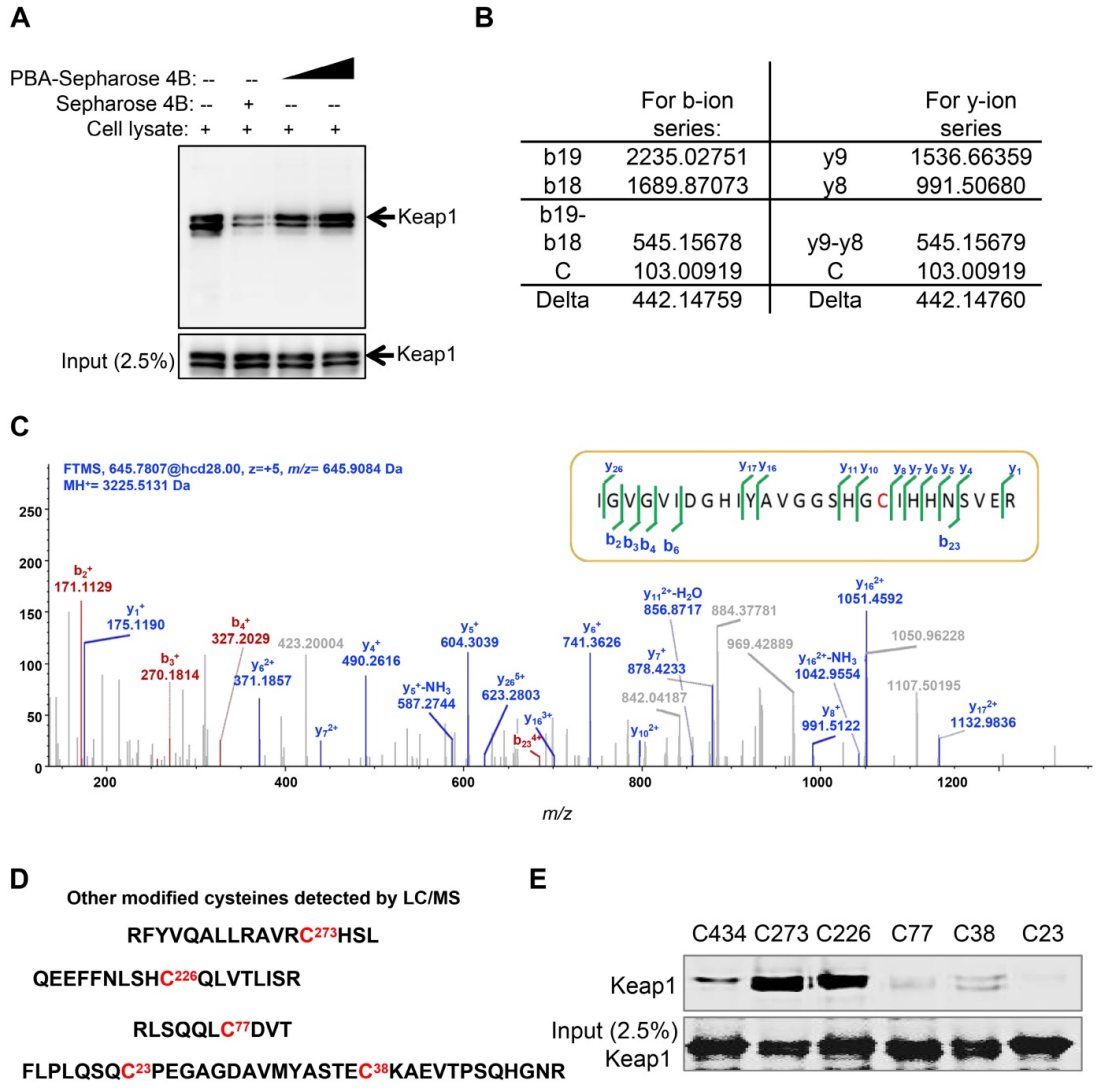
** PBA directly targets Keap1.** (A) Keap1 was pulled down by PBA-Sepharose4B. Total cell lysates were harvested. The lysates were incubated with PBA-Sepharose 4B or Sepharose 4B at 4 °C. After removing the unbound protein, the eluted protein was subsequently detected by immunoblotting for the Keap1 protein. (B and C) Cys434 was covalently modified by PBA. Human His-Keap1 was expressed in BL21 cells. The recombinant Keap1 was incubated with PBA (protein-to-compound molar ratio, 1:20) at 37 °C for 2 h before MS analysis. (D) Other modified cysteines detected by LC/MS/MS. (E) Cys434 and Cys77 of Keap1 were indispensable for its interaction with PBA. The recombinant Keap1 mutant proteins were expressed in BL21 cells and incubated with PBA-Sepharose 4B (200 μl). After removal of the unbound protein, the eluted protein was subsequently detected by immunoblotting for the Keap1 protein.

Keap1 is a cysteine-rich protein and negatively regulates the activation of Nrf2 based on cysteine-induced conformational changes. The Co-IP results showed that PBA significantly disturbed the complex of Keap1 and Nrf2. To evaluate whether PBA could directly bind to intracellular Keap1 and subsequently affect Nrf2 activity, we used a pull-down assay using PBA conjugated Sepharose 4B beads. The result in Figure 5A shows that Keap1 could bind to PBA-Sepharose 4B beads but not bind to Sepharose 4B beads only. Recombinant human Keap1 was purified from* Escherichia coli* BL21 cells for further analyses. To identify the residues that contributed to this interaction, we performed an impartial mass spectrometry (MS) analysis to detect cysteine residues modified by PBA *in vitro*. The MS analysis in Figure 5B-C of chymotrypic peptide covering Cys434 showed an increase of 442.14 in mass, demonstrating that Cys434 was modified by PBA.

In addition to Cys434, covalent modifications of PBA at Cys77, Cys23, Cys38, Cys273 and Cys236 were similarly discovered from the MS study (Figure 5C). Covalent modification of these six cysteines could similarly serve to inactivate Keap1. Thus, we separately mutated these cysteine residues to serine residues to determine which residues were indispensable for binding to PBA. As shown in Figure 5D, mutants of Cys434, Cys77, Cys23 and Cys38 lost the capacity to bind to PBA, while other mutants retained their high binding affinity for PBA. Considering that Cys23 and Cys38 are located in the NTR domain of Keap1 without possible function on Nrf2, we speculated that the two key residues Cys77 and Cys434 are indispensable for its interaction with PBA to regulate the Nrf2 signaling pathway.

### PBA suppresses Keap1-regulated ubiquitination of Nrf2 by disrupting the interaction between Cul3 and Keap1

**Figure 6:**
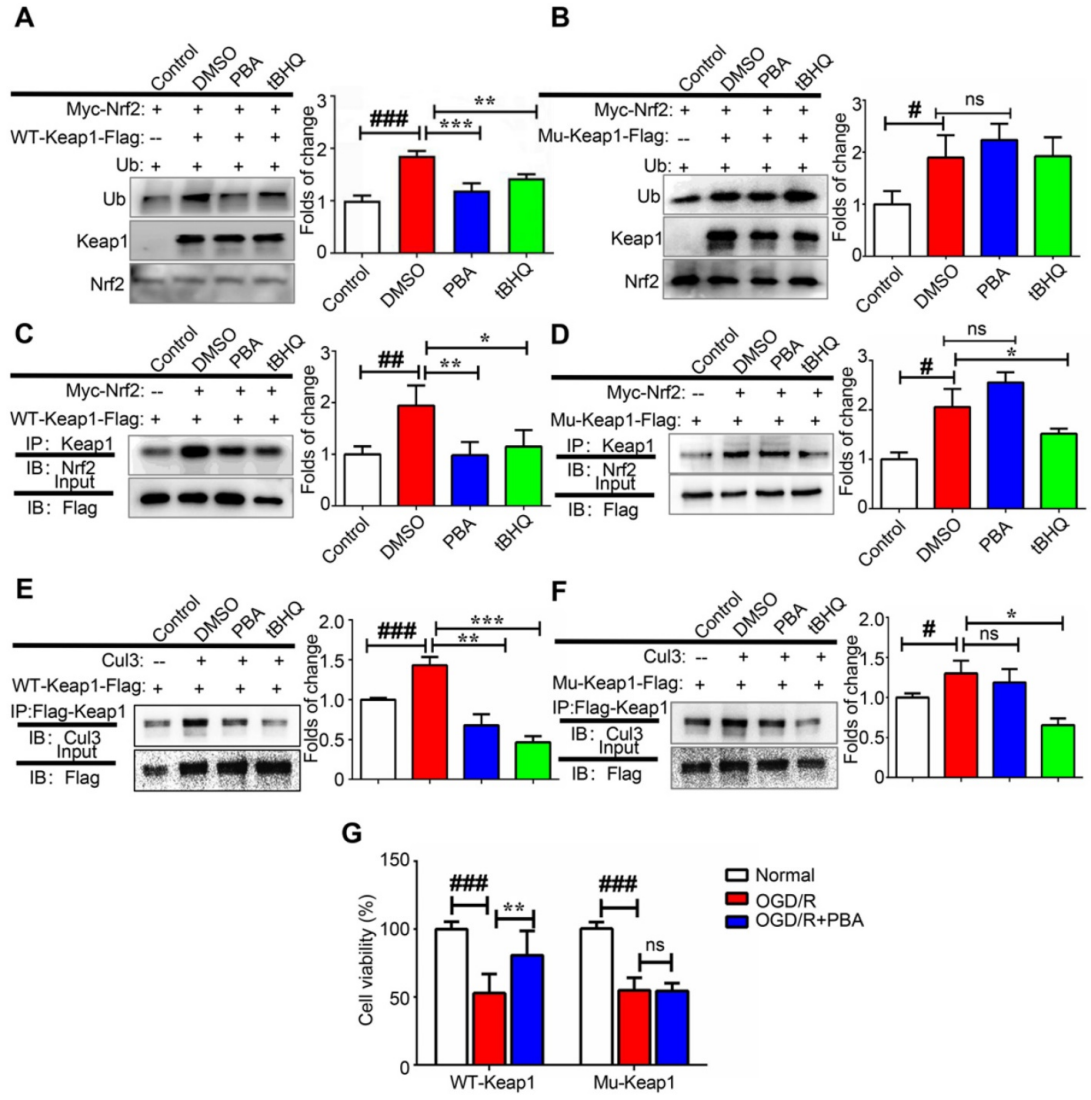
** PBA suppresses Keap1-mediated ubiquitination of Nrf2 by disrupting the interaction between Cul3 and Keap1.** (A and B) PBA suppressed Nrf2 ubiquitination in a Keap1-Cys77 and C434-dependent manner. First, 293T cells were transfected with WT-Keap1 or C77S and C434S-Keap1, Myc-Nrf2, and Ub plasmids for 24 h and treated with 30 μM PBA. The cell lysates were immunoprecipitated using anti-Myc beads, and the ubiquitination level was evaluated using the anti-Ub antibody. (C and D) PBA induced the dissociation of the Nrf2-Keap1 complex. Then, 293T cells were cotransfected with WT-Keap1 and Flag-Nrf2 plasmids for 24 h and treated with 30 μM PBA for 12 h. The cell lysates were immunoprecipitated with the anti-Flag antibody, and the level of Nrf2 was assessed using the anti-Nrf2 antibody. (E and F) PBA disrupted the interaction between Cul3 and Keap1. The 293T cells were transfected with the WT-Keap1 or C77S-C434S-Keap1 plasmids for 24 h and treated with 30 μM PBA for 12 h. The cell lysates were immunoprecipitated with the anti-Flag antibody, and the level of Cul3 was measured using the anti-Cul3 antibody. #*p* < 0.05, ##*p* < 0.01, ###*p* < 0.001, the control vs DMSO; *p < 0.05,**p < 0.01, ***p < 0.001, PBA or tBHQ vs DMSO. (G) C77S and C434-Keap1 mutant blocked the protective effect of PBA against OGD/R-induced primary mouse cardiomyocytes injury. Primary mouse cardiomyocytes were transfected with mouse WT-Keap1 or C77S and C434S-Keap1 mutant vectors. After 24 h, the cells were treated with the indicated doses of PBA and subjected to OGD conditions as described in the Method section for 3 h and under normal conditions for another 18 h. Cell viability was measured by MTT assays. #*p* < 0.05, ###*p* < 0.001, the control vs OGD/R; **p < 0.01, ***p < 0.001, OGD/R+PBA vs OGD/R.

Alkylation of the BTB domain of Keap1 by covalent modifiers contributes to the disruption of the Keap1-Cul3 interactions, leading to Nrf2 activation. Next, we investigated the effect of PBA on the modulation of Nrf2 ubiquitination as Nrf2 stability is controlled by ubiquitin-mediated proteasomal degradation. First, 3X Flag-Keap1, Myc-Nrf2 and Ub plasmids were transfected into 293T cells and then, the cells were treated with 30 μM PBA for 20 h and 5 μM MG132 for another 4 h. Cell lysates were pulled down using anti-Myc beads, and immunopurified Nrf2 was examined for the occurrence of Ub by western blotting. As shown in Figure 6A-B, both PBA and tBHQ significantly suppressed Keap1-dependent Nrf2 ubiquitination. When mutant Cys77S and Cys434S-Keap1 vectors were transfected into 293T cells, PBA was unable to protect Nrf2 from ubiquitination, which indicated that PBA prevented Keap1-driven ubiquitination of Nrf2 in a Cys77-dependent manner. As shown in Figure 6E-F, PBA could disrupt the interaction of Keap1 and Cul3 in WT-Keap1 cells, while the disruption was blocked in the Cys77S and Cys434-Keap1 mutant cells.

### PBA also disturbs the interaction between Keap1 and Nrf2

To investigate the effect of PBA on the interaction of Keap1 and Nrf2, we transfected 293T cells with Flag-Keap1 and Myc-Nrf2 plasmids, and treated them with 30 μM PBA for 24 h. The protein expression of Nrf2 was measured by western blotting analysis followed by immunoprecipitation with Flag beads. As shown in Figure 6C-D, PBA disrupted the interaction between Keap1 and Nrf2, while tBHQ did not affect it. Furthermore, when Cys77S and Cys434S-Keap1 mutant vectors were transfected into 293T cells, PBA was unable to block the Keap1-Nrf2 complex. Therefore, these results suggest that alkylation by PBA could trigger dissociation of the Keap1-Nrf2 complex. Importantly, shown in Figure 5G, Cys77S and Cys434S-Keap1 mutant vectors blocked the protective effect of PBA against OGD/R injury on primary cardiomyocytes.

### Thermodynamic analysis of Keap1 and mutant interactions with PBA

**Figure 7:**
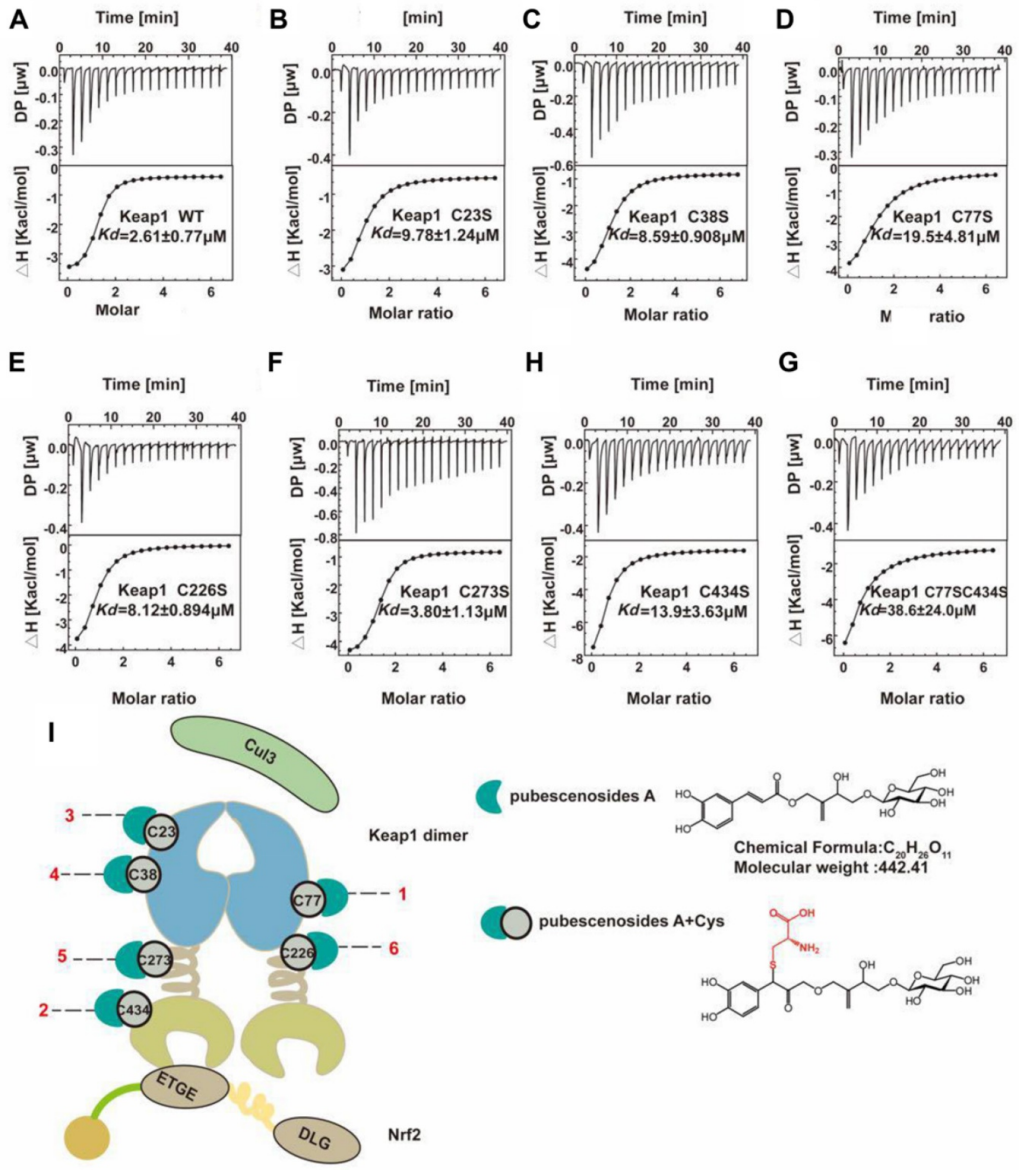
** Representative ITC titration profiles of the titration of PBA with mutation the Keap1 mutant protein.** Keap1-WT (A), Keap1-C23S (B), Keap1-C38S (C), Keap1-C77S (D), Keap1-C226S (E), Keap1-C273S (F), Keap1-C434S (G), Keap1-C77S/C434S (H). The top graphs represent the raw ITC thermograms, and the bottom graphs represent the fitted binding isotherms. The dissociation constant (*K_d_*) is shown below the curves. (I) Schematic representation of the Keap1-PBA interaction order. Keapl is composed of the BTB (white cyan barb), IVR (brown helical), and Keap1-Kelch (Khaki concave) domains. The Keapl homodimer binds with one Neh2 domain of Nrf2 and Cul3. The Cyan crescent represents PBA, the gray-green circle represents cysteine and the number represents the position of cysteine.

Cysteine is an essential amino acid in proteins, and the functional group -SH is a key element in the active centre. Cysteine is the on-off switch for the functions of Keap1, and we demonstrated that the compound PBA derived from herbs can interact with six key cysteines in Keap1. However, the detail mechanism of the binding manner, especially the selection of the combining order with different cysteines is still unclear. From the ITC results shown in Figure 7A-H, (Table 1), we found that PBA had a different preference for cysteines based on the binding constant. The *K_d_* of the interaction between the WT Keap1 and PBA was 2.61 μM, as determined by ITC. Similarly, the *K_d_* values of the mutants Cys77S and Cys434S were 19.5 μM and 13.9 μM respectively. The *K_d_* value of Cys23S, Cys38S, Cys226S and Cys273S were 9.78, 8.59, 8.12 and 3.80 μM, respectively. Collectively, the order in which PBA bound to Keap1 on the cysteines was as follows: Cys77 > Cys434 > Cys23 > Cys38 > Cys226 > Cys273 (Figure 7I), which was consistent with the results from the pull-down assays in Figure 5D. In addition, we constructed the double mutant Cys77S/Cys434S and the *K_d_* was 38.6 μM, which was ~15-fold weaker than that of the WT. These results strongly support the contention that PBA preferentially bound to Cys77 and Cys434.

### The binding mode of PBA with the Keap1-Kelch and Keap1-BTB domains is revealed by covalent docking

**Figure 8:**
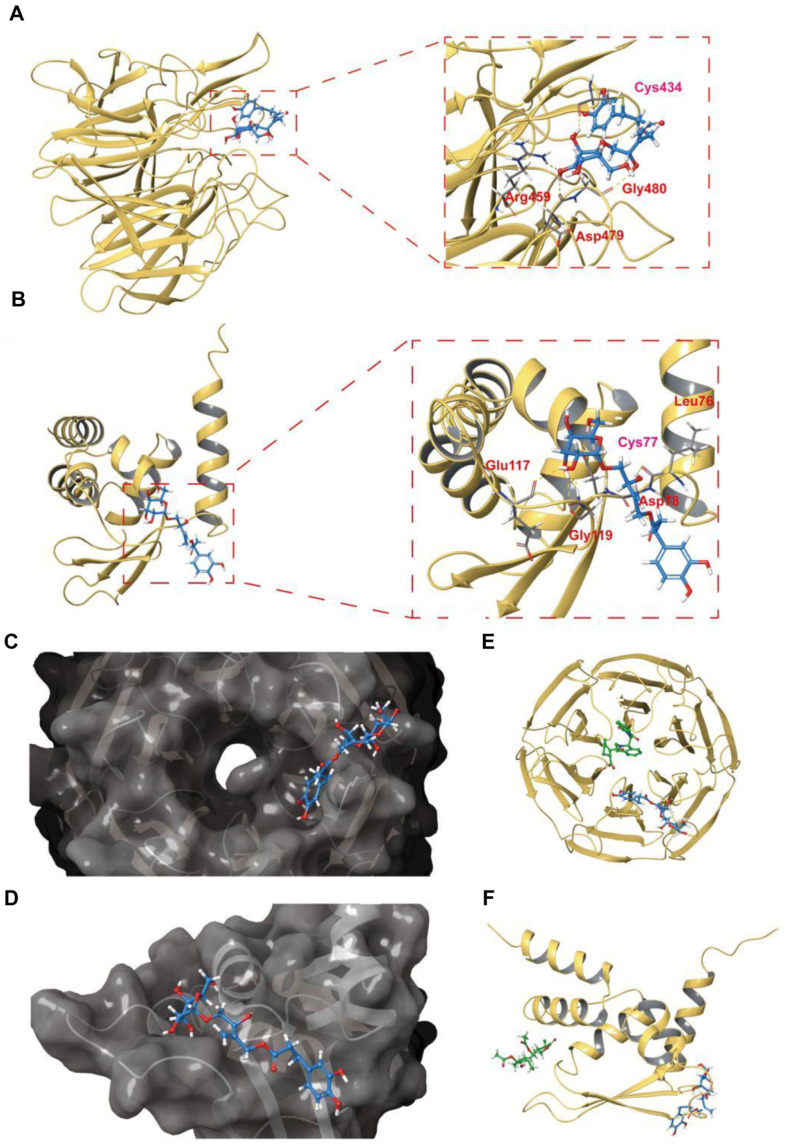
** Docked complex of PBA with the Keap1-Kelch and Keap1-BTB domains.** (A) Structural view of PBA with the Keap1-Kelch domain. (B) The PBA and Keap1-Kelch domain 2D interaction diagram; purple lines represent hydrogen bonds, black lines represent covalent bonds. (C) The surface picture of the Keap1-Kelch domain with PBA bound in the region of Cys434. (D) Details of the interactions between PBA and the Keap1-Kelch domain. The hydrogen bonds are presented as yellow dashed lines. Cys434 of the Keap1-Kelch domain forms a covalent bond with PBA. (E) Structural view of PBA with the Keap1-BTB domain; yellow dashed lines indicate hydrogen bonds. (F) PBA and the Keap1-BTB domain 2D interaction diagram; purple lines represent hydrogen bonds, black lines represent covalent bonds. (G) The surface picture of the Keapl BTB domain with PBA bound in the region of Cys77. (H) Details of the interactions between PBA and the Keapl-BTB domain. The hydrogen bonds are presented as yellow dashed lines. Cys77 of the Keapl-BTB domain forms a covalent bond with PBA.

To reveal the possible interaction mode of PBA and Keap1, we carried out covalent docking studies 26. The docking score ranged from -4.412 to -5.565 for Keap1-Kelch and -1.964 to -4.898 for Keap1-BTB, and the lowest docking score was selected for the following study (Table 2). As shown in Figure 8A-D, the results of covalent docking of PBA with Keap1-Kelch domain revealed that PBA occupied a shallow groove formed by the residues Asn414, Arg380, Asp389, Ser431, Hid432, Gly433, Cys434, Ile135, Hid436, Thr481, Gly480, Asp479, Arg459, Ile461 and Ile435. Among them, PBA interacted hydrophobically with Ser431, His436, Asn414 and Thr481, and the polar interactions were found to be with Gly433 and Gly480.

Hydrogen bonding interactions are also involved in the binding of the Keap1-Kelch domain and PBA. The residues Arg380, Gly480 and Asp479 formed hydrogen bonds with hydroxyl groups with PBA, respectively. Interestingly, Cys434 formed one covalent bond with PBA, and hydrogen bond formed between the nitrogen of Cys434 and the oxygen of PBA, which could enhance the binding affinity between PBA and Cys434. Therefore, the covalent docking results of the Keap1-Kelch domain provided further evidence that PBA directly targeted Keap1 at Cys434 and played a critical role.

In addition, the results of the docking studies in Figure 8E-H showed that PBA interacted hydrophobically with the lipophilic pocket of Keap1-BTB through the Met94, Val98, Met120, Cys77, and Leu76 residues. The polar interactions were found to be with Gln74, Gln75 and Gln118. Moreover, PBA occupied a surface cavity formed by Arg116, Glu 117, Gln118, Gly119, Met120, Gln74, Gln75, Leu76, Cys77, Asp78, His96, Lys97 and Thr80. Importantly, apart from a covalent bond with Cys77, the two adjacent residues Leu76, Asp78 also formed two hydrogen bonds with PBA, Gly119 formed hydrogen bonds with two hydroxyl groups adjacent to PBA, and Gly119 and Glu117 formed hydrogen bonds with the same hydroxyl group. These interaction modes may significantly increase the binding affinity of PBA for the BTB domain.

## Discussion

The NLRP3 inflammasome plays an important role in the pathophysiology of myocardial ischemia reperfusion 19, 27. Suppression of the NLRP3 inflammasome can reduce IL-1β release, subsequently attenuating the inflammatory process and protecting cardiomyocyte death against the ischemia/reperfusion injury 22. Our study found that PBA could significantly inhibit the NLRP3 inflammasome in both macrophages and cardiomyocytes, subsequently reducing the cardiac infarct size. Redox signaling is a critical factor that modulates NLRP3 inflammasomes 28, 29. Decreasing ROS level can block NLRP3 inflammasome activation 30. Nrf2 is a transcription factor and is sensitive to redox potential. This molecule induces antioxidative proteins and antioxidants to suppress ROS 31. Our results showed that PBA could significantly reduce ROS production, promote Nrf2 translocation into nucleus, and upregulate the expression of downstream proteins (HO-1、 NQO1).

Keap1 is a partner of Nrf2. Keap1 could regulate Nrf2 ubiquitination and translocation into nucleus 32. In our present study, we discovered that PBA activated the Nrf2 signaling pathway by covalent modification of Keap1 at cysteine residues. By pull-down assays, we found that PBA directly bound to Keap1. Notably, we used LC/MS/MS and cysteine mutagenesis to show that PBA could bind to Cys77 and Cys434. Given the possible cysteine modifications of PBA, we used an ITC assay to detectthe binding capacity of PBA with WT Keap1 and mutant Keap1. We found that the Cys77S (*K_d_* = 19.5 μM) and Cys434S (*K_d_* = 13.9 μM) mutants showed a lower binding affinity with PBA than the WT (*K_d_* = 2.61 μM). Finally, we found that the order of selection was Cys77 > Cys434 > Cys23 > Cys38 > Cys226 > Cys273, which was consistent with our LC/MS/MS data. From the structure analysis, Cys77 and Cys434 are located in the functional domain for Nrf2 activation, while Cys23 and Cys38 might affect other functions; and we will clarify this issue in future studies.

To gain insight into the differences between PBA and existing inhibitors 33, 34, we revealed the binding mode of PBA with Keap1 by covalent docking (Figure 8). We downloaded six inhibitor complex structures acting on the Kelch domain from PDB for comparison (Figure S3). Our results showed that the main inhibitory site of the traditional inhibitor of the Keap1-Kelch domain is the active site of the central cavity in the Kelch domain, while PBA interacts with the Keap1-Kelch domain on Cys434, which occupies a shallow groove surface. More importantly, PBA forms a covalent bond with Cys434 and produces hydrogen bonding. For the BTB domain, we also downloaded five sets of inhibitors for comparison, such as the inhibitors affecting Cys151. PBA binds to a completely different surface of Keap1. Moreover, we found that both Leu76 and Asp78 play important roles in the helping formation of covalent bonds between Cys77 and Cys434 with PBA.

A small molecule covalently binding to a specific cysteine of Keap1, such as Cys151, Cys273 and Cys288, could activate Nrf2 4, 35. In the present study, we indicated the critical role of Cys77 and Cys434 covalently bonding with PBA in activation of Nrf2. Cys77 is located at the BTB domain for affecting Cul3 and Keap1 interaction, while Cys434 is located at the Kelch domain for regulating Keap1 and Nrf2 interaction. Our results showed that PBA could modify Keap1 by preferentially forming a covalent bond with Cys77 and Cys434. PBA interacted with Cys77 leading to Cul3 dissociation from the Keap1-BTB domain. On the other hand, PBA acting on Cys434 could cause the Keap1 with Nrf2 dissociation easily. Blocking both cysteines could decrease Nrf2 ubiquitination and promote Nrf2 translocation into the cell nucleus. Compared with the traditional Keap1 inhibitor acting on the Keap1-Kelch domain or the Keap1-BTB domain, PBA could not only form stronger covalent bonds, but could also produce two kinds of effects, which is better for preventing drug resistance for Keap1 mutants.

Although there are several Nrf2 activators that directly induce Nrf2 expression or inhibit the interaction of Nrf2 and the Keap1 Kelch domain through a noncovalent mechanism 36, 37, PBA provides a new Nrf2 activator with an accurate Keap1 modification mechanism. In addition, the Keap1-Nrf2 signaling pathway also contributes to detoxification 38. Thus, PBA might be a potential compound with low off-target toxicity and therapeutic effects. As described above, we confirmed that PBA protected the heart against ischemia-reperfusion via the Nrf2 pathway *in vivo* and* in vitro*.

In conclusion, the present study provides primary evidence that PBA improved myocardial ischemia reperfusion injury via suppression of NLRP3 inflammasome activation. We also found that PBA could modify the Cys77 and Cys434 residues of Keap1 to regulate Nrf2 ubiquitination and translocation to the nucleus, subsequently attenuating myocardial ischemia-reperfusion injury. Additionally, the cysteines of Keap1 have different degrees of activation by PBA: Cys77 > Cys434 > Cys23 > Cys38 > Cys226 > Cys273, which gives us deeper insight into the cysteine sensitivity of Keap1.

## Materials and Methods

### Regents

PBA was isolated from* I. pubescens* and identified by Dr. Peng Wu from Guangzhou University of Chinese Medicine. Antibodies against Keap1 (10503-2-AP) was purchased from Proteintech Group, Inc. (Chicago, IL, USA). Glyceraldehyde-3-phosphate dehydrogenase (GAPDH, E12-052), HO-1 (E1A5393), NQO1 (E2A6437), cullin-3 (Cul3, E2A7225V), Flag (E1T508-2) and β-actin (E12-051) antibodies were purchased from Enogene Company (Nanjing, Jiangsu, China). Ubiquitin (Ub, sc-8017), caspase-1 (sc-56036), caspase-1 p20 (sc-398715) and interleukin-1 beta (IL-1β, sc-52012) antibodies were bought from Santa Cruz Biotechnology (Santa Cruz CA, USA). Nrf2 (12721) and lamin B1 (13435) antibodies were purchased from Cell Signaling Technology (Danvers, MA, USA). Nrf2 antibody (ab137550) for immunofluorescence staining was purchased from Abcam (Cambridge, UK). NLPR3 (NBP-2-12446) antibody was purchased from Novus Biologicals, LLC (Centennial, CO, USA). MG-132 (HY-13259) and tBHQ (HY-100489) were purchased from MedChemExpress (Monmouth Junction, NJ, USA).

### Cell culture and treatment

Rat H9c2 cells, human embryonic kidney 293T cells and RAW264.7 cells were purchased from the American Type Culture Collection (Manassas, VA, USA). The cells were cultured in Dulbecco's modified Eagle's medium (DMEM, Gibco, USA) containing 10% foetal bovine serum (FBS, Gibco, USA), 100 U/ml penicillin and 100 μg/mL streptomycin (PS, Gibco, USA). The cells were incubated at 37 °C with 5% CO_2_.

For OGD/R treatment, H9c2 cells were washed twice with phosphate buffered saline (PBS). The medium was changed to glucose and serum free DMEM, and H9c2 cells were incubated in an incubator with 95% N_2_ and 5% CO_2_ at 37 °C. After 6 h, the medium was replaced with DMEM containing 10% FBS and 1% PS. Then H9c2 cells were returned to a normal incubator with 5% CO_2_ balanced with air at 37 °C for the indicated time.

Based on the previous study 15, RAW264.7 cells were treated with different concentrations of PBA 1 h before 200 ng/mL lipopolysaccharide (LPS) stimulation for 6 h. Then, 2 mM adenosine triphosphate (ATP) was added and incubated for 20 min at 37 °C with 5% CO_2_.

### Primary neonatal mouse cardiomyocytes isolation

The heart was removed from the neonatal C57BL/6 mice and cut into 1 mm^3^ pieces in ice PBA buffer. The tissues were digested with the mixture of 0.125% trypsin (Gibco, 15090-046) and 0.1% type II collagenase (Gibco, 17101-015) 6 min at 37 °C for three times until the tissues were completely digested. The cells were harvested and seeded in a dish. After 90 min of incubation, the cell culture medium was collected and centrifuged. The centrifuged cardiomyocytes were harvested, seeded on 0.1 % gelatin-coated culture plates with DMEM containing 10% FBS and 0.1M Brdu, and incubated in a 37°C incubator.

### Cell viability measurement

Cell viability was determined by MTT assays. PBA was prepared to a stock solution dissolved in dimethyl sulfoxide (DMSO). RAW264.7, H9c2 cells, primary mouse cardiomyocytes were treated with different doses of PBA, and DMSO only as control for 48 h for the toxicity. In addition, H9c2 cells or primary mouse cardiomyocytes were treated with the indicated doses of PBA, diazoxide as positive control, DMSO only as control, then cultured with or without OGD/R treatment. At the end of the experiments, MTT was added into each well at the concentration of 0.5 mg/ml, and incubated at 37 °C for 4 h. After removal of the supernatant, 150 μL of DMSO was added to solubilize the formazan products. And the solution was measured at the absorbance of 490 nm by a spectrophotometer (Thermo Fisher Scientific, MA, USA). The results of the MTT assay in all groups were normalized to the control group with DMSO only treatment.

### Intracellular ROS detection

The fluorescent probe DCFH-DA was used to measure intracellular ROS levels. RAW264.7 cells were treated with 100 μg/mL LPS plus ATP as mentioned above. H9c2 cells underwent OGD/R. After the appropriate treatment, the cells were incubated with DCFH-DA at a concentration of 10 μM for 30 min. The cells were washed with PBS three times. A fluorescence microscope and microplate reader were used to measure the level of DCFH-DA fluorescence at the excitation wavelength of 488 nm and the emission wavelength of 525 nm.

### Cell transfection

For transient transfection, HiPerFect Transfection Reagent (Qiagen, German) was used according to the manufacturer's instructions. Nrf2 siRNA (h) (sc-156128) and control siRNA-A (sc-37007) were bought from Santa Cruz Biotechnology. Briefly, H9c2 cells were seeded in 96-well plate at the density of 2x10^4^ cells per well or 12-well plate at the density of 3x10^5^ cells per well. According to preliminary results, transfection with siRNA for 24 h could get a better knockdown effect. Therefore, after transfection with siRNA for 24 h, H9c2 cells were treated with the indicated doses of PBA (which is consistent to the assay above mentioned) before OGD/R insult.

### Western blotting analysis

Total protein was isolated from H9c2 cells, primary mouse cardiomyocytes, RAW264.7 cells and heart tissue using 1X RIPA buffer containing phosphatase and proteinase inhibitors at 4 °C. After measurement of the concentration, the cell lysates were separated by sodium dodecyl sulphate polyacrylamide gel electrophoresis (SDS-PAGE) and transferred to polyvinylidene fluoride (PVDF) membranes. The membranes were blocked in Tris-buffered saline-Tween 20 (TBST) buffer with 5% BSA for 1 h and incubated with primary antibodies (1:1000 dilution) overnight at 4 °C. After the membranes was washed by TBST buffer for three times, they were incubated with horseradish peroxidase (HRP)-conjugated secondary mouse or rabbit antibodies (1:3,000 dilution) at room temperature (RT) for 1.5 h and then detected by SuperEnhanced chemiluminescence (ECL) reaction reagents (GBCBIO Technologies Inc., Guangzhou, Guangdong, China). In brief, drop evenly the ECL working liquid onto the membrane at the ratio of 1 mL ECL working liquid and 10 cm^2^ of membrane. After letting the membranes stand for 1-2 min, the bands of the respective proteins were detected with the exposure time of 10 ~60 s.

### Immunofluorescence staining

According to previous experiences, Nrf2 nucleus translocation could be detected at 6 h. Therefore, briefly, the H9c2 cells were seeded in 15 mm confocal dishes at the density of 2x10^5^ overnight, and incubated with 30 μM PBA for 6 h. RAW264.7 cells were also seeded in in 15 mm confocal dishes at the density of 3x10^5^ overnight and treated with 30 μM PBA for 1 h prior to 200 ng/mL LPS stimulation for 6 h and then induced by 2 mM ATP for another 20 min. After treatment, cells were fixed and incubated with primary antibodies against Caspase-1, ASC, and Nrf2 at 4 °C overnight. After several washes with PBS, the bound antibodies were detected by Alexa Fluor 594-conjugated goat anti-rabbit or Alexa Fluor 488-conjugated goat anti-mouse IgG secondary antibody at RT for 1 h. The cell nuclei were stained with 4', 6-diamidino-2-phenylindole (DAPI, Solarbio Life Science, Beijing, China) at room temperature for 10 min. The cells were examined on a laser scanning microscope (Carl-Zeiss, Jena, Germany). Five or six fields of view per sample were randomly selected to be captured.

### Pull-down assay

PBA-Sepharose 4B beads were created by dissolving PBA in coupling buffer (100 mM NaHCO_3_, pH 8.3, 0.5 M NaCl). After washing with 1 mM HCl medium, Sepharose 4B beads were mixed with PBA coupling buffer and slowly rotated overnight at 4 °C. Excess PBA was washed away with coupling buffer and any remaining active groups were blocked with 0.1 M Tris-HCl buffer (pH 8.0) for 2 h. The medium was then washed with 0.1 M acetate buffer (pH 4.0) containing 0.5 M NaCl followed by a wash with 0.1 M Tris-HCl (pH 8.0) that also contained 0.5 M NaCl. PBA-Sepharose 4B beads were ready for use in the pull-down assay. The RAW264.7 cellular supernatant fraction (600 µg) was incubated with either PBA-Sepharose 4B or Sepharose 4B as a negative control in reaction buffer (50 mM Tris-HCl, pH 7.5, 5 mM EDTA, 150 mM NaCl, 1 mM DTT, 0.01% NP-40, 2 µg/mL of BSA, 0.02 mM PMSF, 1 × protease inhibitor cocktail). After incubation with gentle rocking overnight at 4 °C, the beads were washed five times with wash buffer (50 mM Tris-HCl, pH 7.5, 5 mM EDTA, 150 mM NaCl, 1 mM DTT, 0.01% NP-40, 0.02 mM PMSF) and proteins bound to the beads were analyzed by immunoblotting assay with a Keap1 antibody.

### Animal and drug administration

The experimental procedures and protocols were approved by the Committee on Ethical USE of Animals of International Institute for Translational Chinese Medicine, Guangzhou University of Chinese Medicine (No. IITCM-20180115). Adult male C57BL/6 mice were purchased from the Laboratory Animal Center, Sun Yat-Sen University (Guangzhou, Guangdong, China), and Nrf2 knockout mice were obtained from Riken. Adult male C57BL/6 or Nrf2 knockout mice were treated with 30 mg/kg PBA or an equal volume of vehicle 15 min prior to reperfusion. Mice were divided into 6 groups: (1) Wild-type sham (n = 10): C57BL/6 mice received vehicle, as the sham group; (2) Wild-type AMI/R group (n = 10): C57BL/6 mice received vehicle (i.p.) 15 min before reperfusion; (3) Wild-type AMI/R plus PBA group (n = 10): C57BL/6 mice received 30 mg/kg PBA (i.p.) 15 min before reperfusion; (4) Nrf2 knockout sham (n = 8) : Nrf2 knockout mice received vehicle (i.p.), as in the sham group; (5) Nrf2 knockout AMI/R group (n = 8): Nrf2 knockout mice received vehicle (i.p.) 15 min before reperfusion; (6) Nrf2 knockout AMI/R plus PBA group (n = 8): Nrf2 knockout mice received 30 mg/kg PBA (i.p.) 15 min before reperfusion. PBA was dissolved in 0.9% NaCl buffer containing 2% EtOH. The vehicle was 0.9% NaCl buffer containing 2% EtOH.

### Model of AMI/R

The C57BL/6 mice or Nrf2 knockout mice underwent myocardial ischemia-reperfusion injury as described previously. In brief, mice were anaesthetized by i.p. injection of sodium pentobarbital at a dose of 50 mg/kg and ventilated under a pressure-control VentStar R415 ventilator (RWD Life Science Inc., Shenzhen, China). Following thoracotomy between the 3rd and 4th intercostal spaces, the mouse heart was exposed. The LAD was ligated with an 8-0 silk suture and the PE10 tube for 30 min. PBA and vehicle were i.p. injected 15 min prior to reperfusion. After 30 min of ischemia, the suture with PE10 tube was removed. At the end of 24 h of reperfusion, the LAD was relegated, and 1% Evans blue dye was injected to measure the area at risk (AAR). The hearts of the mice were collected, and frozen at -80 °C for 10 min and cut into 5 slices. The slices were incubated with 1% 2,3,5-triphenyltetrazolium chloride (TTC, Santa Cruz, USA) at 37 °C for 15 min and fixed with 4% paraformaldehyde overnight. The slices were captured and the areas of AAR and IA were quantified by using ImageJ software.

### Hemodynamic measurement

Hemodynamic measurements were performed using a PV-loop catheter (Transonic Systems Inc., Ithaca, NY, USA). Hemodynamic measurements were analysed using Ponemah 7700 modules software according to a standardized protocol. The maximum rate of rise of left ventricular pressure increase (+dp/dt_max_) and the maximum rate of rise of left ventricular pressure decrease (-dp/dt_max_) were calculated for all mice.

### Echocardiographic detection

Echocardiography was performed on mice at the end of reperfusion. In brief, mice were re-anesthetized with isoflurane and subjected to the Vevo 2100 System (VisualSonics Inc., Toronto, Canada). M-mode was used to obtain LV dimensions in diastole and systole according to the standard procedures and calculations. Left ventricular end systolic diameter (LVESD), left ventricular end diastolic diameter (LVEDD), left ventricular end systolic volume (LVSV) and left ventricular end diastolic volume (LVDV) were measured. And the left ventricular ejection fraction (LVEF) and left ventricular fractional shortening (LVFS) values were calculated using Vevostrain software as parameters indicating cardiac function.

### LC/MS/MS detection of modified peptides

Keap1 recombinant protein (1 mg/mL) in PBS buffer was mixed with 50 μM PBA, and incubated at 30 °C for 2 h. After removal of the small molecules by centrifugation, the product was dissolved in 0.1 M Tris-HCl, pH 8.0 containing 8 M urea. Then, 50 mM NH_4_HCO_3_ was added to adjust the pH value. Trypsin was added at a ratio of 1:100. The mixture was incubated in a 37 °C water bath for 16 h. After digestion, the mixture was subjected to a C18 column to remove salt. Finally, the peptides were eluted by the buffer containing 700 μL of 100% ACN, 299 μL of double distilled H_2_O and 1 μL of 0.1% FA. Then, the peptides were analysed by LC/MS/MS.

### Protein cloning, expression and purification

The Keap1 was amplified by PCR using a human cDNA library, and was subcloned into the vector pET28-TEV, which contained one tobacco etch virus (TEV) protease cleavage site at the upstream multiple cloning site. The protein was expressed in *E. coli* BL21 (DE3) cells and incubated at 37 °C until the OD_600_ value reached 0.6, using 0.2 mM isopropyl β-D-1-thiogalactopyranoside (IPTG) overnight induction at 18 °C. The cells were isolated by centrifugation, resuspended in ice-cold lysis buffer and lysed by cryogenic overpressure cell breaker at 1,200 psi. The lysates were centrifuged for 60 min at 14,000 rpm to obtain the supernatant. Gravity flow purification of Keap1 protein was obtained at room temperature using 1 mL of Ni-NTA and eluted in buffer containing 250 mM imidazole, followed by 1 mL of HiTrap QHP for the second step purification. The final protein was purified and validated by Superdex 200 10/300GL resin.

The mutant plasmid DNA was obtained by PCR. The PCR products were cleaved by DpnI restriction enzymes. The restriction endonuclease-cleaved mixture was subsequently transformed into *E. coli* DH5α. Colonies were evaluated by colony PCR and sequenced. The mutant plasmid was transformed into* E. coli* BL21 (DE3) after successful sequencing. The steps for expression and purification of all recombinants were described above.

### ITC titration experiments

Isothermal calorimetry (ITC) titration experiments were performed at 25 °C. WT Keap1, Keap1 mutants and PBA were dissolved in binding buffer (40 mM Tris-HCl pH 8.0, 250 mM NaCl). In a typical experiment, 60 μL of PBA (1 mM) was injected 19 times at 2-min intervals from a stirring syringe into the sample cell containing 300 μL of WT protein or mutants (30 μM). The first injection was 0.4 μL of PBA, and the subsequent 18 injections were 2 μL each time. The site-binding models were determined by using MicroCal Origin software.

### Molecular docking

The receptors Keap1-Kelch and Keap1-BTB were obtained from PDB (PDB ID: 3WN7, 5DAF) in PDB format for docking purposes. Schrodinger software was used for molecular docking. The protein was prepared by assigning bond orders, adding hydrogens, setting proper ionization states of residues, and capping. The protein was then refined with H-bond assignment (water orientations, at neutral pH). The ligands were prepared in ligprep with the following parameters: OPLS3e, do not change ionization states, generate tautomers and stereoisomers (create all combinations of specified chiralities and determine chiralities from 3D structure) with at most 32 ligands to be made. Finally, the ligands were covalently docked by the covalent docking method and the reaction type was selected as Michael addition. The latest best pose for PBA was selected using the docking score.

### Statistics analysis

The results are presented as the mean ± SEM or the mean ± SD from at least three independent experiments. Data analysis was performed using a two-tailed unpaired Student's t test for two-group comparisons. Multiple comparisons were carried out by one-way analysis of variance (ANOVA), followed by Dunnett's test with GraphPad Prism 8 (GraphPad Software, Inc, La Jolla, CA, USA). *P* < 0.05 were considered statistically significant.

## Supplementary Material

Supplementary figures and tables.

## Figures and Tables

**Table 1:** Thermodynamic parameters for binding of PBA to Keap1 mutants at 25 ℃.

Alanine mutation	*K_d_* (μM)	△*H* (kcal/mol)	*T*△*S* (kcal/mol)	△*G* (kcal/mol)
WT	2.61e-6±0.769e-6	-3.31±0.199	4.31±0.00	-7.62±0.00
C23S	9.78e-6±1.24e-6	-3.62±0.241	3.22±0.00	-6.84±0.00
C38S	8.59e-6±0.908e-6	-4.59±0.199	2.32±0.00	--6.91±0.00
C77S	19.5e-6±4.81e-6	-5.65±0.866	0.78±0.00	-6.43±0.00
C226S	8.12e-6±0.894e-6	-5.11±0.279	1.84±0.00	-6.95±0.00
C273S	3.80e-6±1.13e-6	-3.87±0.271	3.53±0.00	-7.40±0.00
C434S	13.9e-6±3.63e-6	-4.76±0.367	1.87±0.00	-6.63±0.00
C77S/C434S	38.6e-6±24.0e-6	-4.92±0.637	1.11±0.00	-6.03±0.00

*K_d_* value is kind of the dissociation constant and △*H*, *T*△*S*, △*G* are the change in binding enthalpy, entropy, and Gibbs energy, respectively. -*RT* In*K_d_* =△*G* =△*H*-*T*△*S*, where *T* and* R* are the absolute temperature and gas constant, respectively. Values are shown as means ± standard deviations from triplicate runs.

**Table 2:** The docking scores of Keap1-Kelch, Keap1-BTB with PBA.

Domain	Docking score				
Keap1-Kelch	-4.412	-5.565	-4.656	-4.652	-5.436
Keap1-BTB	-1.964	-2.963	-4.898	-2.907	-3.925

The docking score was obtained from the project table after docking completed. Red numbers are the lowest docking score and selected it for study.

## Abbreviations

Keap1: Kelch ECH-associating protein 1
Nrf2: nuclear factor erythroid 2-related factor 2
MI/R: myocardial ischemia-reperfusion
PBA: pubescenosides A
LAD: left anterior descending artery
Cys77: Cysteine 77
Cys434: Cysteine 434
PIDD: protein with a death domain
ROS: reactive oxygen species
IL-1β: interleukin-1β
HO-1: heme oxygenase-1
NQO1: NAD(P)H quinone dehydrogenase 1
Cul3: Cullin-3
Ub: ubiquitin
FBS: fetal bovine serum
PS: penicillin and streptomycin
PBS: phosphate buffered saline
LPS: lipopolysaccharide
ATP: adenosine triphosphate
HRP: horseradish peroxidase
RT: room temperature
ECL: enhanced chemiluminescence
DAPI: 4', 6-diamidino-2-phenylindole
TTC: 2,3,5-triphenyltetrazolium chloride
AAR: area at risk
IPTG: isopropyl β-D-1-thiogalactopyranoside
ITC: isothermal calorimetry
ANOVA: analysis of variance
