# Supplementary material for: Covalent modification of Keap1 at Cys77 and Cys434 by pubescenoside a suppresses oxidative stress-induced NLRP3 inflammasome activation in myocardial ischemia-reperfusion injury
Source: Theranostics. 2021 Jan 1;11(2):861–77. doi: 10.7150/thno.48436 (PMC7738871; doi:10.7150/thno.48436)
Supplement: Supplementary file 1 — Supplementary figures and tables. [file thnov11p0861s1.pdf]

Supplementary data

Covalent modification of Keap1 at Cys77 and Cys434 by pubescenoside A suppresses oxidative stress-induced NLRP3 inflammasome activation in myocardial ischemia-reperfusion injury

Yuanyuan Cheng#, Liangkai Cheng#, Xiang Gao, Sixuan Chen, Peng Wu, Caiyan Wang, Zhongqiu Liu

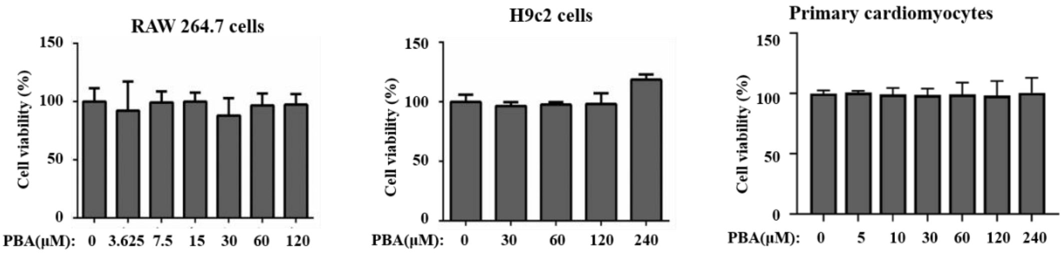

Figure S1. The cytotoxicity of PBA on RAW264.7, H9c2 cells and primary cardiomyocytes. Cells were seed in 96-well plates, and treated with indicated concentrations of PBA. After 48 h, the cell viability was measured by MTT assays.

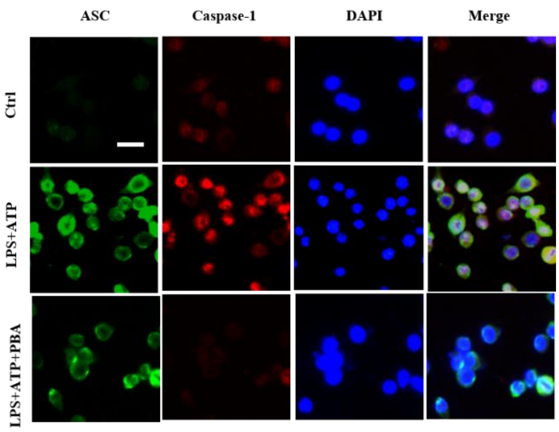

Figure S2. Immunofluorescent staining of NLRP3 inflammasome in LPS plus ATP-induced RAW264.7 macrophages.

Figure S3.

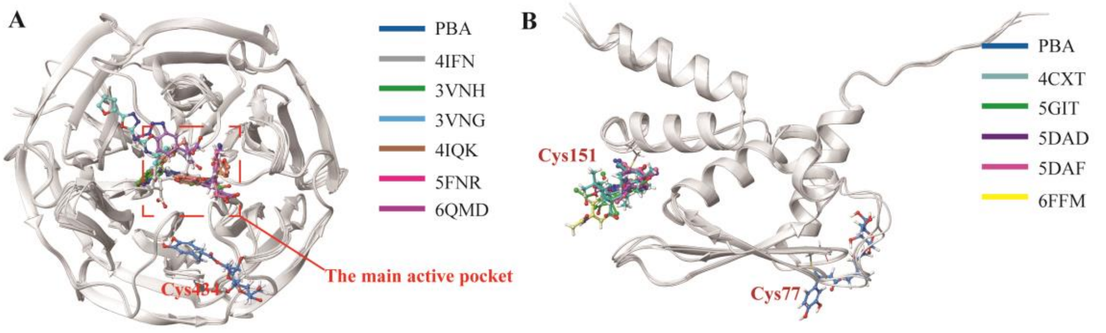

Figure S4. Compare of the difference between PBA and Keap1-Kelch, Keap1-BTB complexes with other compounds. (A) Alignment of PBA and Keap1-Kelch complexes with other six compounds. Navy blue carbon skeleton representation of PBA. The compound of gray carbon skeleton PDB ID: 4IFN, the compound of green carbon skeleton PDB ID: 3VNH, The compound of light blue carbon skeleton PDB ID: 3VNG. The compound of brown carbon skeleton PDB ID: 4IQK. The compound of magenta carbon skeleton PDB ID: 5FNR. The compound of purple carbon skeleton PDB ID: 6QMD. (B) Alignment of PBA and Keap1-BTB complexes with other five compounds. The compound of steelblue carbon skeleton PDB ID: 4CXT, The compound of green carbon skeleton PDB ID: 5GIT, The compound of purple carbon skeleton PDB ID: 5DAD, The compound of magenta carbon skeleton PDB ID: 5DAF, The compound of yellow carbon skeleton PDB ID: 6FFM.

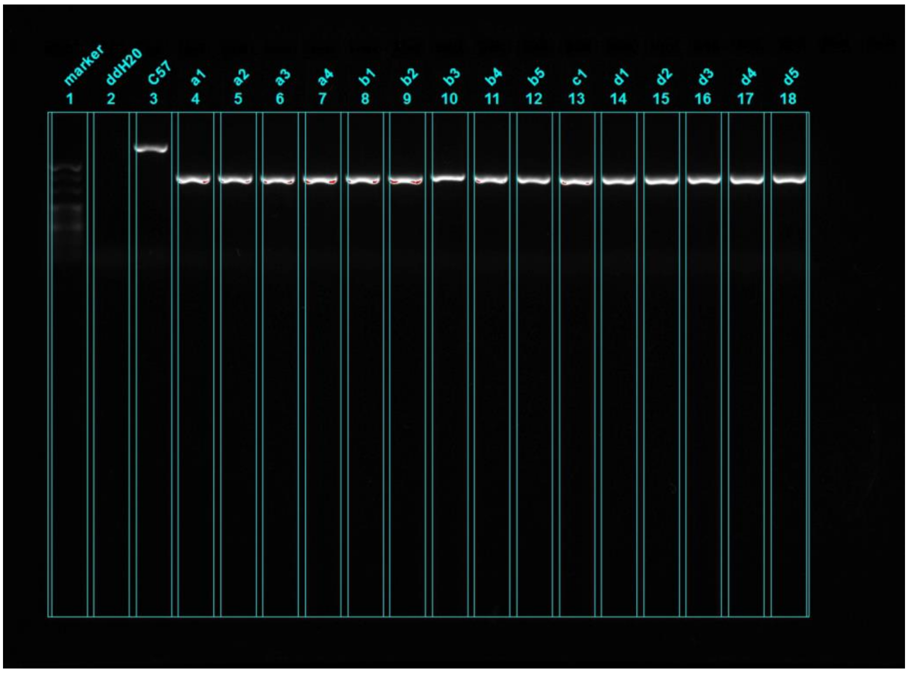

Figure S5. Animal genotyping Nrf2$^{-/-}$

Table S1 MS data for modification peptides of Keap1.

Master	Accession	Description	Exp. q-value	Sum PEP Score	Coverage	# Peptides	# PSMs	# Unique Peptides	# Protein Groups	# AAs	MW [kDa]	calc. pI	Score Sequest HT	# Peptides Sequest HT
Master Protein	Q14145	Kelch-like ECH-associated protein 1 OS=Homo sapiens OX=9606 GN=KEAP1 PE=1 SV=2	0	1169.415	92	67	1690	67	1	624	69.6	6.44	2216.53	67
Confidence	**Sequence**	**Modifications**	**Qvality PEP**	**Qvality q-value**	**# Protein Groups**	**# Proteins**	**# PSMs**	**Master Protein Accessions**	**# Missed Cleavages**	**Theo. MH+ [Da]**	**XCorr Sequest HT**	**Confidence Sequest HT**	**Percolator q-Value Sequest HT**	**Percolator PEP Sequest HT**
High	FLPLQSQCPEGAGDAVM EVTPSQHGNR	2xP+K [C8; C23]	0.221107	0.006789	1	1	1	Q14145	1	4606	0.68	High	0.000286	0.003679
High	CVLHVMNGAVMYQIDS	2xOxidation [M6; M11]	0.013046	0	1	1	3	Q14145	0	2166.05	2.25	High	0	5.49E-06
High	NNSPDGNTDSSALDCYN CAPMSVPR	2xOxidation [M19; M29]	0.030908	0	1	1	8	Q14145	0	3601.472	1.4	High	0	3.7E-05
High	SGLAGCVVGGLLYAVG	1xP+K [C6]	0.00071	0	1	1	3	Q14145	0	2091.032	2.92	High	0	9.62E-09
High	LNSAECYYPER	1xP+K [C6]	0.132884	0	1	1	3	Q14145	0	1786.736	1.2	High	0.000286	0.002801
High	EYIYMHFGEVAKQEEFF TLISR	1xP+K [C22]	0.108654	0	1	1	2	Q14145	1	4073.919	1.29	High	0	0.000651
High	ACSDFLVQQLDPSNAIGI CVELHQR	1xP+K [C2]	0.317631	0.006789	1	1	1	Q14145	0	4028.889	0.89	High	0.000286	0.009711
High	IGVGVIDGHIYAVGGSH ERYEPER	1xP+K [C19]	0.004547	0	1	1	2	Q14145	1	3899.829	2.11	High	0	5.46E-07

High	IGVGVIDGHIYAVGGSHER	1xP+K [C19]	0.014371	0	1	1	21	Q14145	0	3225.527	2.88	High	0.000286	0.006206
High	IYVLGGYDGHTFLDSVEWSEVTR	1xP+K [C18]	3E-05	0	1	1	2	Q14145	0	3995.722	3.32	High	0	1E-11
High	QEEFFNLSHCQLVTLISR	1xP+K [C10]	0.016185	0	1	1	4	Q14145	0	2606.233	1.95	High	0	8.82E-06
High	CVLHVMNGAVMYQIDS	1xP+K [C1]	0.000878	0	1	1	3	Q14145	0	2576.208	2.46	High	0	1.53E-08
High	FLPLQSQCPEGAGDAVMEVTPSQHGNR	1xP+K [C]	0.000446	0	1	1	20	Q14145	1	4163.852	3.55	High	0	1.45E-08
High	AREYIYMHFGEVAK	1xOxidation [M7]	0.206335	0.006789	1	1	2	Q14145	1	1729.837	1.53	High	0.000286	0.003086
High	QAFGIMNELR	1xOxidation [M6]	0.002041	0	1	1	12	Q14145	0	1194.594	2.13	High	0	9.57E-08
High	EYIYMHFGEVAK	1xOxidation [M5]	0.005929	0	1	1	9	Q14145	0	1502.698	2.92	High	0	1.09E-06
High	SGAGVCVLHNCIYAAGG[illegible]SVERYDVETETWTFVAPM	1xOxidation [M43]	0.001405	0	1	1	6	Q14145	1	4810.186	2.83	High	0	4.24E-08
High	EQGMEVVSIEGIHPKVM	1xOxidation [M4]	0.002269	0	1	1	1	Q14145	1	2184.079	4.02	High	0	1.2E-07
High	EQGMEVVSIEGIHPK	1xOxidation [M4]	9.22E-05	0	1	1	20	Q14145	0	1668.826	3.51	High	0	1.29E-07
High	AMFTNGLR	1xOxidation [M2]	0.067597	0	1	1	6	Q14145	0	925.456	1.92	High	0	0.000216
High	FLPLQSQCPEGAGDAVM	1xOxidation [M17]; 2xP+K [C8; C23]	0.037	0	1	1	1	Q14145	1	4621.99	1.68	High	0	5.65E-05

	EVTPSQHGNR		384							4				
High	FLPLQSQCPEGAGDAVM EVTPSQHGNR	1xOxidation [M17]	0.000 798	0	1	1	8	Q14145	1	3737.69 9	3.14	High	0	1.24E-08
High	YDVETETWTFVAPMK	1xOxidation [M14]	6.15E -05	0	1	1	28	Q14145	0	1832.84 1	2.94	High	0	6.39E-10
High	YEPERDEWHLVAPMLTR	1xOxidation [M14]	5.9E- 05	0	1	1	25	Q14145	1	2158.03 9	4.3	High	0	4.34E-11
High	IFEELTLHKPTQVMPCRA	1xOxidation [M14]	0.174 098	0.0067 89	1	1	1	Q14145	1	2354.23 6	1.36	High	0.000286	0.002022
High	LIEFAYTASISMGEK	1xOxidation [M12]	0.109 135	0	1	1	6	Q14145	0	1675.82 5	1.57	High	0	0.000657
High	CHSLTPNFLQMQLQKCEI	1xOxidation [M11];1XP+K (C16)	0.065 175	0	1	1	3	Q14145	1	2835.35 9	2.46	High	0.000286	0.005691
High	YQDAPAAQFMAHK	1xOxidation [M10]	0.000 186	0	1	1	15	Q14145	0	1493.68 4	3.43	High	0	8.3E-06
High	YQDAPAAQFMAHKVVL	1xOxidation [M10]	0.256 904	0.0067 89	1	1	1	Q14145	1	2608.32 3	1.13	High	0.000286	0.005449
High	NNSPDGNTDSSALDCYN CAPMSVPR	1xOxidation [M]	0.000 481	0	1	1	22	Q14145	0	3585.47 7	2.49	High	0	1.39E-06
High	MITAMNTIR	1xOxidation [M]	0.004 77	0	1	1	17	Q14145	0	1066.53 8	2.48	High	0	1.6E-05
High	CVLHVMNGAVMYQIDS	1xOxidation [M]	0.004 547	0	1	1	2	Q14145	0	2150.05 6	1.09	High	0	5.48E-07

Table S2. MS/MS analysis of peptides (IGVGVIDGHIYAVGGSHGCIHHNSVERYEPER).

#1	b⁺	b²⁺	b³⁺	b⁴⁺	b⁵⁺	Seq.	y⁺	y²⁺	y³⁺	y⁴⁺	y⁵⁺	#2
1	114.09134	57.54931	38.70196	29.27829	23.62409	I						27
2	171.11280	86.06004	57.70912	43.53366	35.02838	G	3112.44298	1556.72513	1038.15251	778.86620	623.29442	26
3	270.18122	135.59425	90.73192	68.30076	54.84206	V	3055.42151	1528.21439	1019.14536	764.61084	611.89012	25
4	327.20268	164.10498	109.73908	82.55613	66.24636	G	2956.35310	1478.68019	986.12255	739.84373	592.07644	24
5	426.27110	213.63919	142.76188	107.32323	86.06004	V	2899.33163	1450.16946	967.11540	725.58837	580.67215	23
6	539.35516	270.18122	180.45657	135.59425	108.67685	I	2800.26322	1400.63525	934.09259	700.82126	560.85847	22
7	654.38210	327.69469	218.79889	164.35098	131.68224	D	2687.17916	1344.09322	896.39790	672.55025	538.24165	21
8	711.40357	356.20542	237.80604	178.60635	143.08653	G	2572.15221	1286.57975	858.05559	643.79351	515.23626	20
9	848.46248	424.73488	283.49234	212.87108	170.49832	H	2515.13075	1258.06901	839.04843	629.53814	503.83197	19
10	961.54654	481.27691	321.18703	241.14209	193.11513	I	2378.07184	1189.53956	793.36213	595.27342	476.42019	18
11	1124.60987	562.80857	375.54147	281.90793	225.72780	Y	2264.98777	1132.99753	755.66744	567.00240	453.80338	17
12	1195.64698	598.32713	399.22051	299.66720	239.93522	A	2101.92445	1051.46586	701.31300	526.23657	421.19071	16
13	1294.71540	647.86134	432.24332	324.43431	259.74890	V	2030.88733	1015.94730	677.63396	508.47729	406.98329	15
14	1351.73686	676.37207	451.25047	338.68967	271.15319	G	1931.81892	966.41310	644.61116	483.71019	387.16960	14
15	1408.75833	704.88280	470.25763	352.94504	282.55749	G	1874.79745	937.90237	625.60400	469.45482	375.76531	13
16	1495.79035	748.39882	499.26830	374.70305	299.96389	S	1817.77599	909.39163	606.59685	455.19946	364.36102	12
17	1632.84927	816.92827	544.95461	408.96777	327.37567	H	1730.74396	865.87562	577.58617	433.44145	346.95461	11
18	1689.87073	845.43900	563.96176	423.22314	338.77997	G	1593.68505	797.34616	531.89987	399.17672	319.54283	10
19	2235.02751	1118.01740	745.68069	559.51234	447.81132	C-P+K	1536.66359	768.83543	512.89271	384.92135	308.13854	9
20	2348.11158	1174.55943	783.37538	587.78335	470.42814	I	991.50680	496.25704	331.17379	248.63216	199.10718	8
21	2485.17049	1243.08888	829.06168	622.04808	497.83992	H	878.42274	439.71501	293.47910	220.36114	176.49037	7
22	2622.22940	1311.61834	874.74798	656.31281	525.25170	H	741.36383	371.18555	247.79279	186.09641	149.07859	6
23	2736.27233	1368.63980	912.76229	684.82354	548.06029	N	604.30491	302.65610	202.10649	151.83169	121.66680	5
24	2823.30436	1412.15582	941.77297	706.58155	565.46669	S	490.26199	245.63463	164.09218	123.32095	98.85822	4

25	2922.37277	1461.69002	974.79577	731.34865	585.28038	V	403.22996	202.11862	135.08150	101.56295	81.45181	3
26	3051.41536	1526.21132	1017.80997	763.60930	611.08889	E	304.16155	152.58441	102.05870	76.79584	61.63813	2
27						R	175.11895	88.06311	59.04450	44.53520	35.82961	1
